# ASO Author Reflections: Blood Group as a Guide for Adjuvant Chemotherapy in Pancreatic Cancer

**DOI:** 10.1245/s10434-025-17381-y

**Published:** 2025-04-30

**Authors:** Yosuke Inoue, Manabu Takamatsu, Yohei Masugi, Tsuyoshi Hamada, for the GTK Pancreatic Cancer Study Group in Japan

**Affiliations:** 1 Department of Hepatobiliary and Pancreatic Surgery, Cancer Institute Hospital, Japanese Foundation for Cancer Research, Tokyo, Japan; 2 Division of Pathology, Cancer Institute, Japanese Foundation for Cancer Research, Tokyo, Japan; 3 Department of Pathology, Cancer Institute Hospital, Japanese Foundation for Cancer Research, Tokyo, Japan; 4 Department of Pathology, Keio University School of Medicine, Tokyo, Japan; 5 Division of Diagnostic Pathology, Keio University School of Medicine, Tokyo, Japan; 6 Department of Gastroenterology, Graduate School of Medicine, The University of Tokyo, Tokyo, Japan; 7 Department of Hepato-Biliary-Pancreatic Medicine, Cancer Institute Hospital, Japanese Foundation for Cancer Research, Tokyo, Japan

## Past

Given the poor prognosis of patients with resected pancreatic cancer, there is an urgent need for biomarkers to guide adjuvant treatment selection beyond carbohydrate antigen 19-9. The ABO blood group has been linked to the inherent risk of pancreatic cancer.^1^ However, few studies have investigated its prognostic role and interaction with adjuvant chemotherapy regimens in patients with resected pancreatic cancer.^2,3^ Furthermore, there have been only limited data linking blood group antigen expression in pancreatic cancer cells to survival outcomes.^4^

## Present

In a large multicenter study within the GTK Pancreatic Cancer Consortium (*n* = 1153),^5^ the ABO blood group was not associated with disease-free or pancreatic cancer-specific survival. However, these associations differed by adjuvant chemotherapy regimen (*P*_interaction_ < 0.012). In a Cox proportional hazards regression model for patients receiving no adjuvant chemotherapy, blood groups A, B, and AB were associated with multivariable-adjusted hazard ratios for disease-free survival (95% confidence interval) of 0.99 (0.69–1.41), 1.65 (1.09–2.48), and 1.79 (1.01–3.17), respectively (vs. O). Among patients receiving S-1-based chemotherapy, blood group AB exhibited a reversed association (multivariable hazard ratio, 0.63; 95% confidence interval, 0.39–1.00; vs. O). In stratified analyses with reversed groups, the superiority of S-1-based chemotherapy over gemcitabine-based chemotherapy was evident only in patients with blood groups A and AB. In an immunohistochemical analysis of blood group antigens in pancreatic cancer cells, aberrant expression (i.e., inconsistent with the patient’s blood group) was uncommon, and an interaction between the expression status of these antigens and the adjuvant chemotherapy regimens was consistently observed.

## Future

These findings have clinical and research implications for improving outcomes in patients with resected pancreatic cancer. First, the underlying mechanism by which the ABO blood group influences differential survival outcomes across adjuvant chemotherapy regimens should be elucidated. If validated by functional studies, this knowledge could aid in discovering novel biomarkers and therapeutic targets. Second, the potential role of the ABO blood group in guiding treatment selection should be further explored, particularly in the context of other adjuvant chemotherapy regimens (e.g., FOLFIRINOX) or neoadjuvant chemotherapy.
